# Congestion … Sinister … Vena Amoris … and the LOVE ♥ of Microsurgery

**DOI:** 10.1055/a-2802-3156

**Published:** 2026-05-29

**Authors:** Joon Pio Hong, Marco Innocenti, Geoffrey G. Hallock

**Affiliations:** 1 Department of Plastic and Reconstructive Surgery, Asan Medical Center, University of Ulsan College of Medicine, Seoul, Republic of Korea; 2 Department of Plastic Surgery, Rizzoli Institute, University of Bologna, Bologna, Italy; 3 Division of Plastic Surgery, St. Luke's Hospital, Sacred Heart Division, Allentown, Pennsylvania, United States

**Figure:**
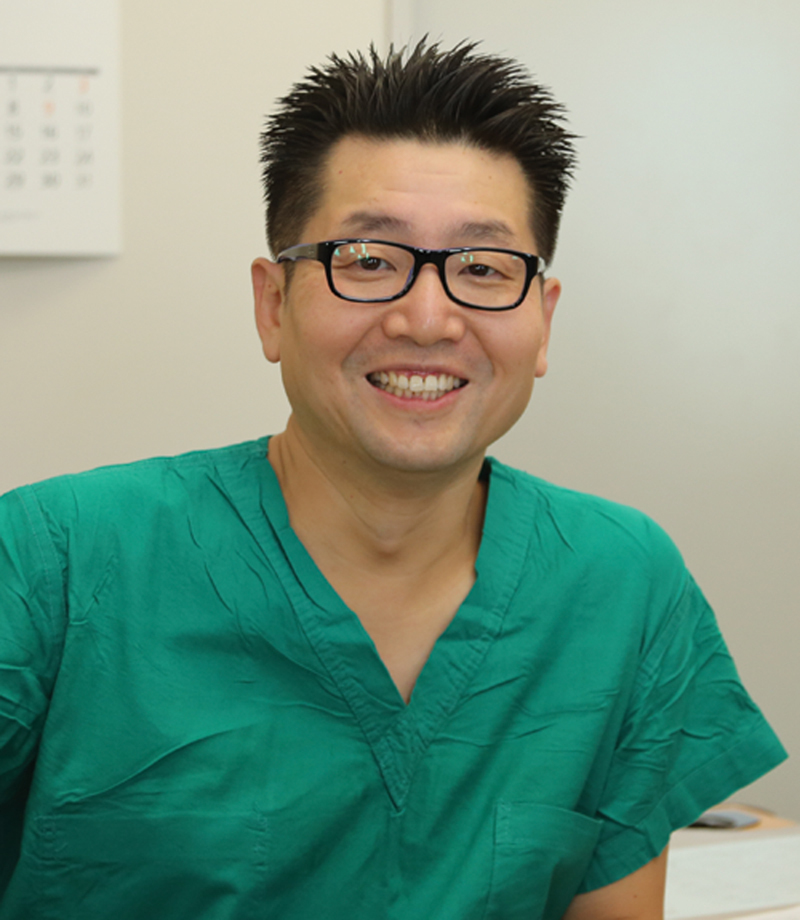
Joon Pio Hong, MD, PhD, MMM (Editor Emeritus of APS)

**Figure:**
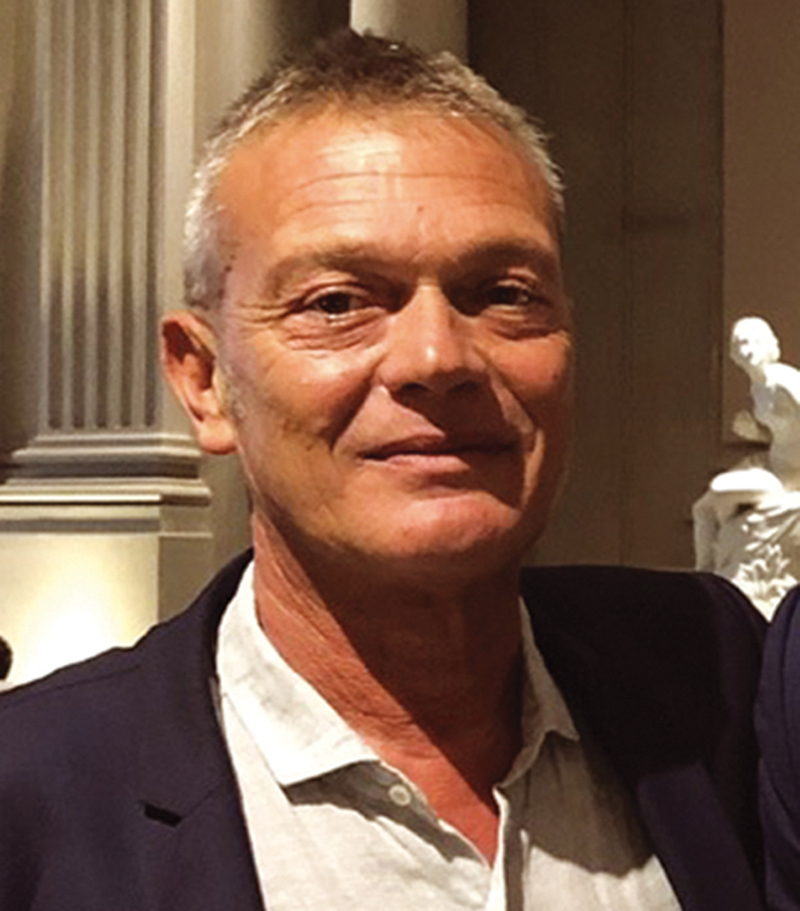
Marco Innocenti, MD

**Figure:**
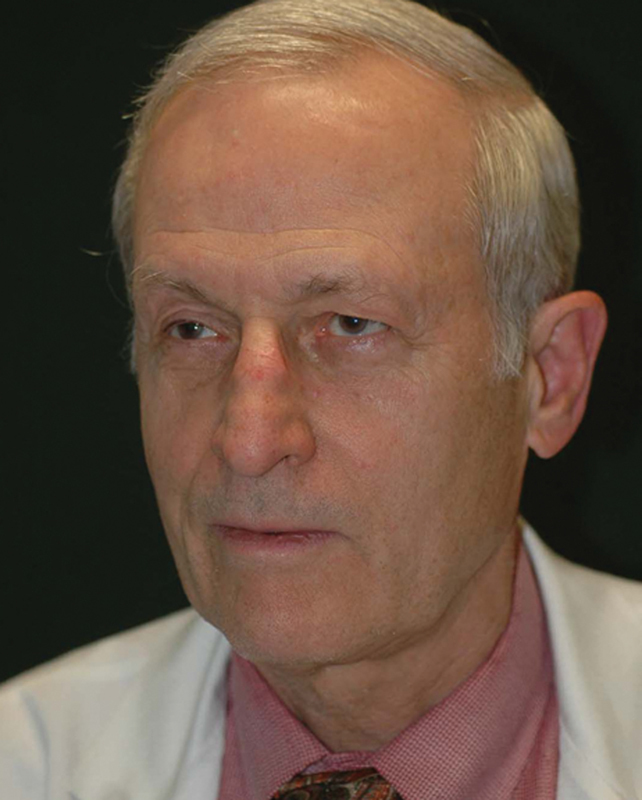
Geoffrey G. Hallock, MD

*Don't write your name on the sand, waves will wash it away.*

*Don't write your name in the sky, the wind may blow it away.*

*Write your name inside the hearts of people you come in touch with.*

*That's where it will stay. 1*

*Quel che sará sará; Que Sera, sera; Quidquid erit, erit* —“what will be, will be.” That *“dead”* language Latin is very much “ *alive* ,” merely peruse the human anatomy to see, a basic skill set by destiny a forte of all “true” plastic surgeons. Latin cannot be sidestepped, 2 so germane to our history as we witness here by following one narrow pathway where the dilemma of venous congestion has been chosen since often thrust upon us all, whether we be cosmetic or reconstructive surgeon. The anguish of purplish discoloration, rapid capillary refill or blue blood on pinprick forewarns distress, whether the situation be breast reduction, facelift, propeller flap, digit replantation, or so much more commonly in our experience the free tissue transfer. Yet our pain and agony are anything but new. Esser's “ Biological or Artery Flaps . . . ” a century or more ago stated that “ *A free transplanted flap would have a better chance of living than a flap with artery intact and veins and branches definitively destroyed* .” 3 Barron 4 later purposely inflicted venous congestion on tubed pedicles to estimate the adequacy of flap circulation.

Commiserating so often within our world of microvascular surgery, the threat of venous compromise, whether called “venous suffering,” “venous insufficiency,” or simply “venous complication,” is omnipresent. 5 Plan ahead to minimize the risk, be ready for venous supercharging, 6 prophylactic venous augmentation, 7 vein–patch interpositions, 8 or ordinary vein grafts 9 —ultimately seeking the superior recipient site, and the list goes on ad infinitum without an escape. When desperate, nonsurgical methods for venous offloading are sought when the repeated anastomotic revision is not successful nor practical. So many possibilities, take, for example, the ever-expanding role of negative pressure wound therapy. 10 But of all, only hirudotherapy has historically proven efficacy, as a leech actively offloads blood from congested tissue while passively initiates improvement via numerous biological mechanisms. 5

The aforementioned leech most assume is a lesser creature (e.g., the segmented worm of the phylum Annelida, Hirudo medicinalis 11 ), but in Ancient times was directly an appellation for doctors themselves, as they had a habit of sticking these creatures onto patients for the “beneficial” effects of bloodletting. 12 Usually, the fourth finger next to the little of the nondominant hand was where attached, perhaps in part explaining why that digit became the “leechman finger,” if not called other names such as the “physic or physician finger,” or even sometimes the “nameless finger.” 12 Indeed, all these machinations might convey the word *sinister* —something evil, dangerous, or threatening. Yet in Latin is not right handed “dexter or dextrous,” i.e., skillful? If “ambo” = both, surgeons simultaneously also using their paralyzed hand would be “ *ambidextrous* ” or right-handed on both sides. What then remains to be only left-handed, is it not also “ *sinister;* ” and therefore meant to be unlucky, to die younger, have more diseases, and even be considered inept 13 —perhaps characterizing the elder of this trio?

**Fig. 1:**
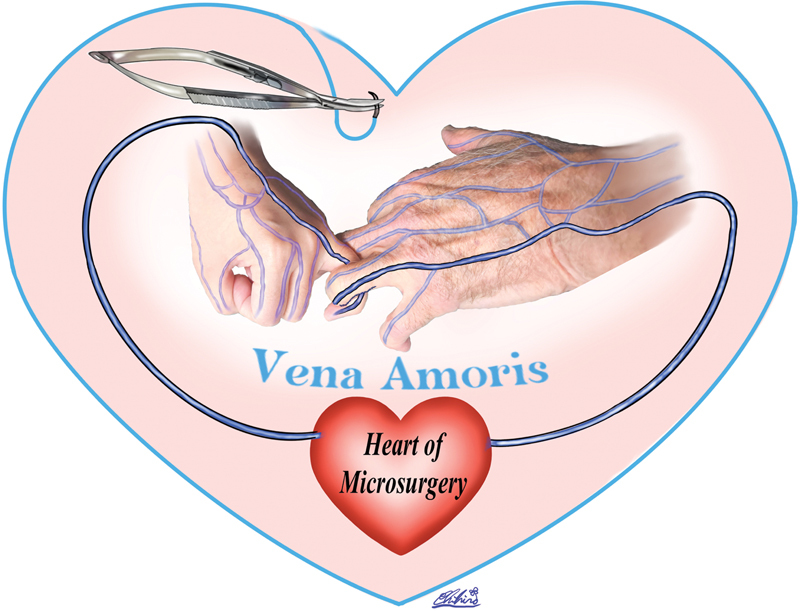
The vena amoris, the vein of each ring finger here interlocked, return directly to the heart of microsurgery, each understandably “a vein of love.”

So now more understandably the “leechman finger,” since sinister, usually involved the fourth finger of the left nondominant hand. Forget not that finger was presumed to be even in Egyptian times a ready source of blood via a vein there that flowed directly to the heart. 14 15 The ecclesiastical lawyer Swinburne, posthumously it has been said, published a work in 1686 that was the earliest known occurrence of the Latin phrase that called the interconnections of this vein the vena amoris ( Fig. 1 ), which in a somewhat romantic fashion would be translated the “vein of love.” 15 16 Thus, the legend if true, that finger should be encircled with a crown to honor it, 15 or instead a wedding ring as Swinburne suggested “ *as they give their hands each to other, so likewise they should give their hearts* ,” thereby explaining why in many Western countries today this may still be bestowed on what now is termed the ring finger . 14 15

Time moves on—dogma again inaccurate as we know now blood returns to the heart from all fingers, not just the sinister ring finger, that functions primarily only as a stabilizer. 16 But the fear of venous congestion ever remains sinister, persistently demanding a solution that we as plastic surgeons must find. Do we not constantly seek *perfection* , while being both *patient* and *pragmatic* ; and yet always a little bit *paranoid* 17 —while anticipating that a venous complication will happen as it invariably does to all of us? Instead, seek that perfect vein, and when found will be so loved—indeed a vena amoris. During this search for the best, never forget to reach out to your teammates, as together no matter what the liability encountered may then only be overcome. At present concede the singular “amo, amas, amat,”—I love, you love, he/she/it loves—far better always the plural “amamus”—we all love—only together will our better outcome make as all truly “aesthetic surgeons.” Chirurgiae microscopicus amoris = the “love of microsurgery,” our love but far more our ***PASSION*** .
